# Design and performance analysis of low damage anti-skid crescent drills for bone drilling

**DOI:** 10.1186/s13018-024-04983-7

**Published:** 2024-08-17

**Authors:** Jing Zhao, Di Wu, Xiaojie Wu, Ziyang Zhang, Zhiguo Wen, Sinan Liu

**Affiliations:** 1 Department of Stomatology, Beijing Tongren Hospital, Capital Medical University, Beijing, 100730 China; 2 School of Control and Mechanical Engineering, Tianjin Chengjian University, Tianjin, 300384 China

**Keywords:** Bone drilling, Skid resistance, Drilling Force, Temperature

## Abstract

**Background:**

With orthopedic surgery increasing year on year, the main challenges in bone drilling are thermal damage, mechanical damage, and drill skid. The need for new orthopedic drills that improve the quality of surgery is becoming more and more urgent.

**Methods:**

Here, we report the skidding mechanism of drills at a wide range of inclination angle and propose two crescent drills (CDTI and CDTII). The anti-skid performance and drilling damage of the crescent drills were analyzed for the first time. Inclined bone drilling experiments were carried out with crescent drills and twist drills and real-time drilling forces and temperatures were collected.

**Results:**

The crescent drills are significantly better than the twist drill in terms of anti-skid, reducing skidding forces, thrust forces and temperature. The highest temperature is generated close to the upper surface of the workpiece rather than at the hole exit. Finally, the longer crescent edge with a small and negative polar angle increases the rake angle of the cutting edge and reduces thrust forces but increases skidding force and temperature. This study can promote the development of high-quality orthopedic surgery and the development of new bone drilling tools.

**Conclusion:**

The crescent drills did not skid and caused little drilling damage. In comparison, the CDTI performs better in reducing the skidding force, while the CDTII performs better in reducing the thrust force.

## Introduction

Bone drilling is a common procedure used in various orthopedic surgeries, including fracture fixation, artificial joint replacement, and core decompression [1]. Cortical bone possesses unique properties such as high strength, anisotropy, semi-brittleness [2]. And the thermal conductivity of cortical bone is low [3]. The drilling thermo-mechanical effect can cause irreversible damage to the bone due to the complex material properties of cortical bone, such as thermal necrosis, fracture, and micromechanical damage [4]. These damages affect the bone tissue surrounding the hole, reducing the quality of the surgery, and affecting the patients’ postoperative recovery.

The main challenges in bone drilling include thermal damage, mechanical damage, drill breakage and drill skidding, as reviewed by Lee et al. [5]*.* Excessive temperatures can lead to thermal necrosis of bone tissue. The threshold for thermal osteonecrosis is 47 °C lasting for 1 min [6]. Excessive thrust forces and torques will cause bone cracks, resulting in loosening of implants, and even breaking of drills, which will cause surgical failure. In addition, due to the irregular surface of the bone, the drill is prone to skid during surgery. This can result in unnecessary bone tissue damage, drill breakage and a reduction in the accuracy of the hole. Facing these challenges, the design of novel orthopedic drills is a good way to control the thrust force, temperature, and drill skid during bone drilling procedures.

Forces and temperatures during bone drilling have been investigated by many scholars. Sui et al. [7] developed a mechanistic model of the cutting lips, the outer portion of the chisel edge and the inner portion of the chisel edge of twist drills (TDs) to predict the thrust force and torque for bone drilling operation. Liu et al. [8] investigated the distribution of the rake angle of cutting edges and established a mechanistic model applicable to different drill geometries to predict the thrust force and torque. Kabiri and Talaee [9] proposed an analytical model of heat propagation in bone drilling based on the hyperbolic Pennes bioheat transfer equation and conducted experiments on bovine cortical bone drilling. The results showed that thermal necrosis extends from 1 to 10 mm around the hole surface. To date, researchers have studied bone drilling in a relatively single way, lacking a comprehensive analysis of bone drilling forces, temperature, damage, and drill skid, and proposing solutions. Drill geometry affects the force and temperature of bone drilling and improving the drill design is one of the most feasible and cost-effective solutions to mitigate bone thermo-mechanical damage [10]. Soriano et al. [11] reported that the rake angle, margin width and body thinning influence the force and temperature in bone drilling, with the rake angle having the greatest effect. Liao et al*.* [12] conducted bone orthogonal cutting experiments to assess cutting stresses at different osteon-tool relative engagement angles (osteon orientation) and uncut chip thickness. Zhang et al. [4] found that drill geometry, flute number, step number and especially chisel edge strongly influence on bone damage. In addition, they emphasized that the overall drilling performance should be considered when evaluating the superiority or inferiority of drills rather than relying solely on drilling force.

On this basis, several scholars have conducted research on novel drills. Soldatos et al. [11] conducted human tibia drilling experiments using straight and tapered drills. Their main conclusions are that (i) the drill diameter has a great effect on temperature, while drilling speed has little effect on temperature and (ii) tapered drills generate significantly generate more heat. Finite element modelling is an effective method [13]. Akhbar and Yusoff [14] employed finite element modelling and response surface methodology for multi-objective optimization of the surgical drill. A novel drill with the point angle of 124.9°, helix angle of 30° and web thickness of 5% has been proposed based on the optimization. Experimental results showed that the optimized drill reduced drilling temperature and the diameter of thermal osteonecrosis. Feldmann et al. [15] designed a single flute drill with a high rake angle (35°) and a short chisel edge. They reported that compared with the two flutes drill, the single flute drill is more effective in chip removal and is less prone to clogging of bone chips. As a result, the drilling temperature decreased. Most of the previous research on novel drills focused on reducing bone drilling temperature and thermal damage. However, the design of orthopedic drills with excellent comprehensive performance is still lacking. Undeniably, orthopedic drills with the ability to reduce drilling forces and temperature and inhibit drill skid are in high demand.

In the realm of materials processing, few scholars have studied the mechanism of drill skidding and novel anti-skid drill bits. Gong et al. [16] proposed motion models for drill deflections and reported that drill skidding stems from unbalanced radial forces resulting from drill installation errors, grinding errors, dislocation errors and other factors such as surface roughness and machine vibrations. These will only cause a slight non-perpendicularity of the drill to the workpiece, so they do not consider the effect of the inclination angle of the workpiece on skid. And they calculated the dynamic chip area but did not investigate the effect of drill geometry on it to guide the design of a new anti-skid drill [17]. Shu et al. [18] also developed a dynamic chip area model and introduced a three-step drill structure which reduced the mechanical and thermal damage and improved the position accuracy in bone drilling. Bai et al. [19] proposed a similar three-step drill and compared its drilling performance with a variety of commercial medical drills. The results showed that the proposed three-step drill can achieve lower thrust force, reduced bending force, smaller temperature rise and higher hole quality. These three-step drills enable anti-skid at small inclination angles. However, due to the lack of outer straight cutting edges and margins, the roundness of the machined hole is not accurate enough. Furthermore, the dynamic chip area models described above are all only suitable for small inclination angles. Meanwhile, according to clinician feedback, bone drilling at large inclination angles is often required because of the uncertainty of the surgical position. Therefore, there is a need to model drill skidding over a wide range of inclination angles and the new design of the drill must have excellent anti-skid properties.

In this paper, we report the design of two novel crescent drills and their anti-skid and damage reduction for the first time. An analytical model for skid at an inclination angle of 0°-60° is developed and the effects of crescent edge length and polar angle on thrust forces, skidding forces and temperature are analyzed. In addition, observations of hole wall temperature during drilling were made to find where the highest temperature occur. The comprehensive drilling performance of the proposed drills is satisfactory, and the crescent drills have potential for clinical applications. This study will also inspire the development of new orthopedic drills in the future.

## Materials and methods

### Analysis of drill skid

**Fig. 1:**
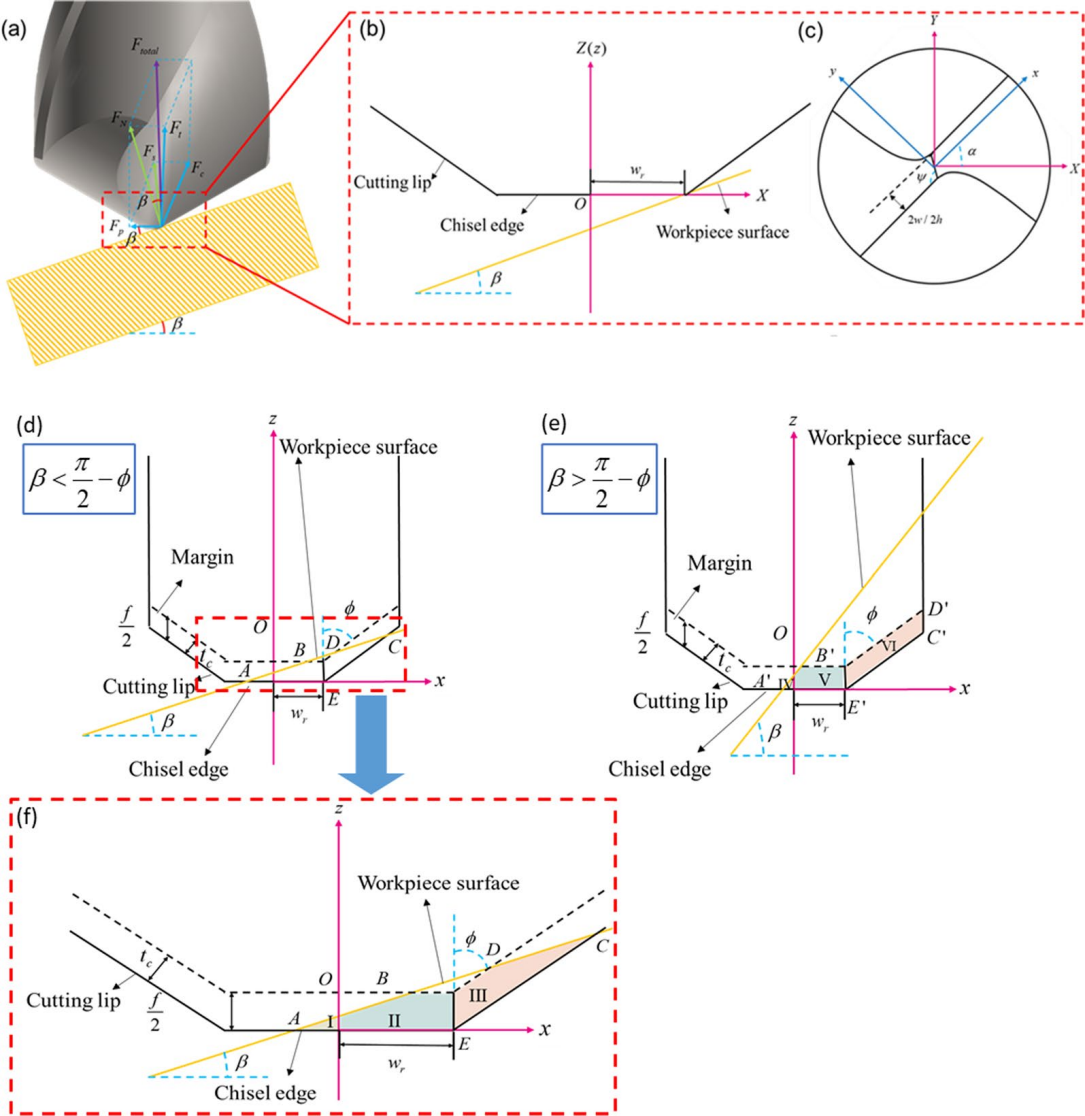
Analysis of drill skid **a** schematic diagram of drilling forces **b** fixed coordinate system **c** rotational coordinate system **d** chip area difference when $\beta < \frac{\pi }{2} - \phi$ **e** Chip area difference when $\beta > \frac{\pi }{2} - \phi$ **f** A detailed view of (**d**)

Because the surface of the bone is irregularly curved, in parctice the drill and the bone surface are often not perpendicular or even heavily inclined, which will result in skid of the drill. As shown in Fig. 1a, when drilling at an incline, the total cutting force at a point on the cutting edge is $F_{total}$, which can be decomposed into a thrust force $F_{t}$ directed vertically upwards, a radial force $F_{p}$ pointing towards the drill axis and a cutting force $F_{c}$ in the opposite direction to the cutting velocity, where both $F_{p}$ and $F_{c}$ are in the horizontal plane. In vertical drilling, this force $F_{p}$ will not arise, however, in inclined drilling, due to the asymmetry of the cutting on both sides, the radial forces cannot be cancelled out and skid tends to occur. The resultant force of $F_{p}$ and $F_{t}$ is the workpicec support force $F_{N}$ on the drill. *F*_*N*_ is perpendicular to the workpiece surface. The angle between *F*_*N*_ and *F*_*P*_ is 90°−*β*. Therefore, *F*_*N*_ act at *β* with respect to *F*_*t*_. According to the geometric relationships in Fig. 1a, $F_{p} = F_{N} \sin \beta$, where $\beta$ is the inclination angle of the workpiece. It can be observed that as $\beta$ increases, $F_{p}$ increases, leading to more severe skid.

At the moment when the drill tip touches the workpiece, a fixed coordinate system is established with the XYZ direction of the dynamometer, using the drill tip as the origin, and the Z-axis is co-linear with the spindle, as shown in Fig. 1b. The asymmetry of the chip area on both sides due to inclined drilling is an important cause of skid. The following analysis shows the asymmetric chip area when drilling at an incline.

With the drill tip as the origin, a rotating coordinate system *xyz* is established, with the *z*-axis co-linear with the Z-axis, the *x*-axis parallel to the cutting lips and the *y*-axis perpendicular to the *x*-axis and the *z*-axis. The *xyz* coodinate system rotates and feeds with the drill, as shown in Fig. 1c. $\alpha$ is the rotaion angle of the drill, which can be expressed by the following equation.

1 $$\alpha = \omega t$$

where *t* is the cutting time (s), $\omega$ is the angular velocity (rad/s) and the value of $\omega$ can be calculated by

2 $$\omega = \frac{\pi N}{{30}}$$

where *N* is the spindle speed (r/min).

Thus, according to the geometric relationship, the two coordinate systems can be transformed by [18]

3 $$\left( {\begin{array}{*{20}c} X \\ Y \\ Z \\ \end{array} } \right) = \left[ {\begin{array}{*{20}c} {\cos \alpha } & { - \sin \alpha } & 0 \\ {\sin \alpha } & {\cos \alpha } & 0 \\ 0 & 0 & 1 \\ \end{array} } \right]\left( {\begin{array}{*{20}c} x \\ y \\ z \\ \end{array} } \right) + \left( {\begin{array}{*{20}c} 0 \\ 0 \\ { - V_{f} t} \\ \end{array} } \right)$$

where $V_{f}$ is the feed speed (mm/s),

4 $$V_{f} = \frac{fN}{{60}}$$

where *f* is the feed rate (mm/r).

As shown in Fig. 1b, the expression for the workpiece surface is

5 $$Z_{w} = X\tan \beta - w_{r} \tan \beta$$

where $w_{r}$ is half the length of the chisel edge. And $w_{r} = w/\cos (\psi - \pi /2)$, where $\psi$ is the chisel edge angle and 2*w* is the core thickness. By transforming the coordinates, a time-dependent expression for the workpiece surface can be obtained:

6 $$z = (x\cos \alpha - y\sin \alpha )\tan \beta - w_{r} \tan \beta + V_{f} t$$

There are two typical cases of drill entry. As shown in Fig. 1d, e, when $\beta < \frac{\pi }{2} - \phi$, point E touches the workpiece surface first, and the chisel edge and cutting lip are involved in cutting. Conversely, when $\beta > \frac{\pi }{2} - \phi$, point C′ touches the workpiece surface first and the cutting lip and margin are involved in cutting, where $2\phi$ is the point angle of the drill. The unsharpness of the marigin can also lead to more severe skid. Figure 1d, e shows the chip area for half a cycle (one cut with a single cutting edge).

As shown in Fig. 1d, the equation of the chisel edge in the rotating coordinate system is

7 $$z = 0, \, - w_{r} \le x \le w_{r} .$$

The coordinates of the intersections A and A′ betweet the chisel edge and the workpiece surface can be obtained by combining Eqs. (11) and (12).

8 $$\left\{ {\begin{array}{*{20}c} {x_{A} = x_{{A^{\prime } }} = \frac{{w_{r} \tan \beta - V_{f} t}}{(\cos \alpha + \sin \alpha \tan \psi )\tan \beta }} \\ {z_{A} = z_{{A^{\prime } }} = 0} \\ \end{array} } \right..$$

Then the coordinates of the intersections B and B′ between the chisel edge and the workpiece surface at the previous cut (half a cycle ago) are

9 $$\left\{ {\begin{array}{*{20}c} {x_{B} = x_{{B^{\prime } }} = \frac{{w_{r} \tan \beta - V_{f} \left( {t - \frac{T}{2}} \right)}}{(\cos \alpha + \sin \alpha \tan \psi )\tan \beta }} \\ {z_{B} = z_{{B^{\prime } }} = 0} \\ \end{array} } \right.$$

where *T* is the rotation period of the drilling.

The equation for the cutting lip $l_{EC}$ can be obtained from the geometric relationships in Fig. 1d.

10 $$z = \left( {x - w_{r} } \right)\cot \phi , \, w_{r} \le x \le R$$

where *R* is the radius of the drill. Similar to point A, it is combined with the equation for the workpiece surface and the coordinates of their intersection C is solved.

11 $$\left\{ {\begin{array}{*{20}c} {x_{C} = \frac{{w_{r} (\cot \phi - \tan \beta ) + V_{f} t - w\sin \alpha \tan \beta }}{\cot \phi - \cos \alpha \tan \beta }} \\ {z_{C} = \left[ {\frac{{w_{r} (\cot \phi - \tan \beta ) + V_{f} t - w\sin \alpha \tan \beta }}{\cot \phi - \cos \alpha \tan \beta } - w_{r} } \right]\cot \phi } \\ \end{array} } \right..$$

Then the coordinates of the intersection D between the cutting lip and the workpiece surface at the previous cut is

12 $$\left\{ {\begin{array}{*{20}c} {x_{D} = \frac{{w_{r} (\cot \phi - \tan \beta ) + V_{f} \left( {t - \frac{T}{2}} \right) - w\sin \alpha \tan \beta }}{\cot \phi - \cos \alpha \tan \beta }} \\ {z_{D} = \left[ {\frac{{w_{r} (\cot \phi - \tan \beta ) + V_{f} \left( {t - \frac{T}{2}} \right) - w\sin \alpha \tan \beta }}{\cot \phi - \cos \alpha \tan \beta } - w_{r} } \right]\cot \phi } \\ \end{array} } \right.$$

Based on the geometric relationships in Fig. 1d, the area of Zone I can be obtained:

13 $$A_{{c{\text{I}}}} = \frac{{\left( {w_{r} \tan \beta - V_{f} t} \right)^{2} }}{{2(\cos \alpha + \sin \alpha \tan \psi )^{2} \tan \beta }}$$

The area of Zone II is:

14 $$A_{{c{\text{II}}}} = \frac{{fw_{r} }}{2} - \frac{{\left[ {w_{r} \tan \beta - V_{f} \left( {t - \frac{T}{2}} \right)} \right]^{2} }}{{2(\cos \alpha + \sin \alpha \tan \psi )^{2} \tan \beta }}$$

The area of Zone III is:

15 $$A_{{c{\text{III}}}} = \frac{{\left[ {2w_{r} (\cot \phi - \tan \beta ) + V_{f} \left( {2t - \frac{T}{2}} \right) - 2w\sin \alpha \tan \beta } \right]f}}{{4\left( {\cot \phi - \cos \alpha \tan \beta } \right)}} - \frac{{fw_{r} }}{2}$$

The area of Zone IV is:

16 $$A_{{c{\text{IV}}}} = \frac{{\left( {w_{r} \tan \beta - V_{f} t} \right)^{2} }}{{2(\cos \alpha + \sin \alpha \tan \psi )^{2} \tan \beta }}$$

The area of Zone V is:

17 $$A_{{c{\text{V}}}} = \frac{{fw_{r} }}{2} - \left[ {\frac{{w_{r} \tan \beta - V_{f} \left( {t - \frac{T}{2}} \right)}}{(\cos \alpha + \sin \alpha \tan \psi )\tan \beta }} \right]\left[ {\frac{f}{4} - \frac{{w_{r} \tan \beta - V_{f} t}}{2(\cos \alpha + \sin \alpha \tan \psi )}} \right]$$

The area of Zone VI is:

18 $$A_{{c{\text{VI}}}} = \frac{f}{2}(R - w_{r} )$$

Therofore, the chip area difference on both sides of the drill in the two cases is

19 $$\Delta A_{{c{\text{a}}}} = A_{{c{\text{II}}}} + A_{{c{\text{III}}}} - A_{{c{\text{I}}}}$$

20 $$\Delta A_{{c{\text{b}}}} = A_{{c{\text{V}}}} + A_{{c{\text{VI}}}} - A_{{c{\text{IV}}}}$$

**Fig. 2:**
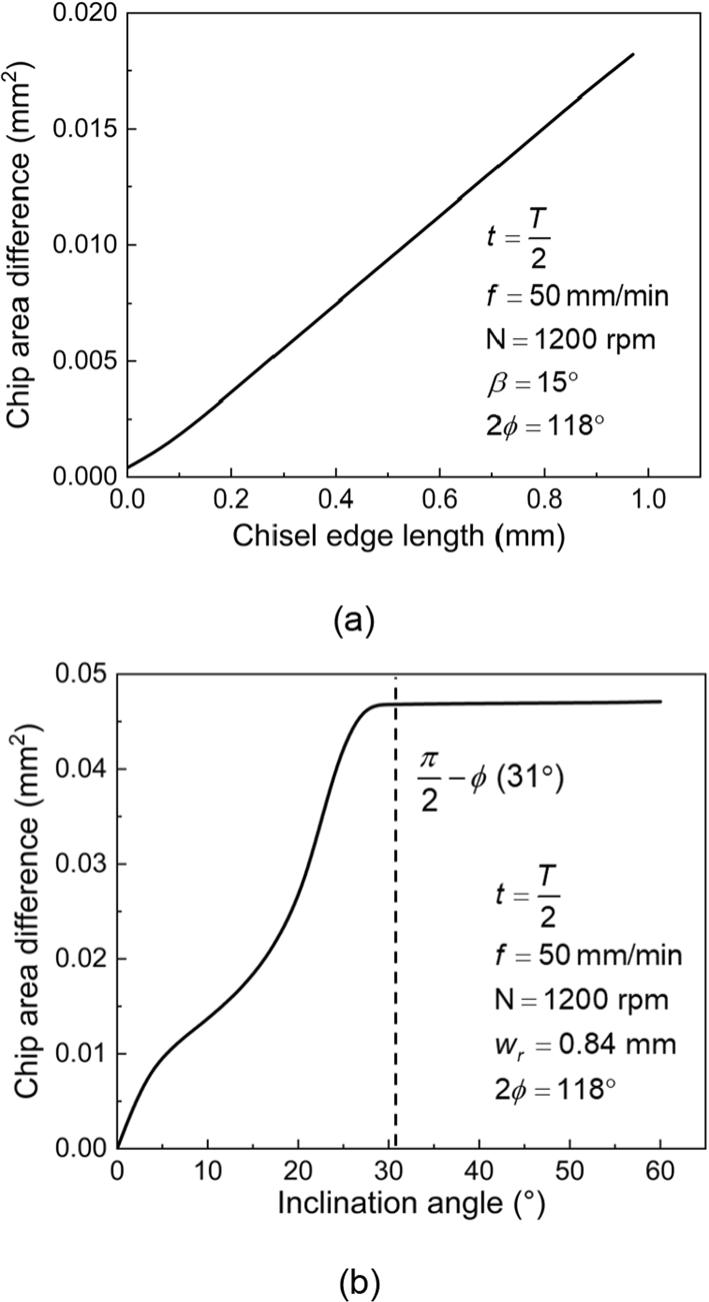
**a** Relationship between the chisel edge length and the chip area difference **b** relationship between the inclination angle and the chip area difference.

Based on these relationships, it is possible to depict the effect of the chisel edge length and the drilling inclination angle on the chip area difference, as shown in Fig. 2. The diameter of the drill used for analysis is 4.5 mm and the cutting time* t* is half a period after the midpoint of the chisel edge has engaged in the cutting, similar to Fig. 1d, e. In this study, the point angle of drills $2\phi$ is 118° ($\frac{\pi }{2} - \phi$ is 31°). It can be seen from Fig. 2a that the chip area difference increases almost linearly with the increasing chisel edge length. In Fig. 2b, as the inclination angle increases, the chip area difference increases significantly when the inclination angle $\beta$ is small. When the inclination angle $\beta$ is large, the chip area difference changes little. This is because when the inclination angle is large ($\beta > \frac{\pi }{2} - \phi$), the margin touches the workpiece first and the cutting lip on one side is fully engaged in cutting before the chisel edge enters the workpiece, as shown in Fig. 1e. At this point the chip area difference is almost at its peak, while continuing to increase the inclination angle does not change the cutting property, so the chip area difference remains at a high level. Thus, from the perspective of tool design, the chisel edge needs to be shortened. The area difference analysis performed for the twist drill provides the basis for the improvement of tool structure.

### Proposal of the crescent drill

**Fig. 3:**
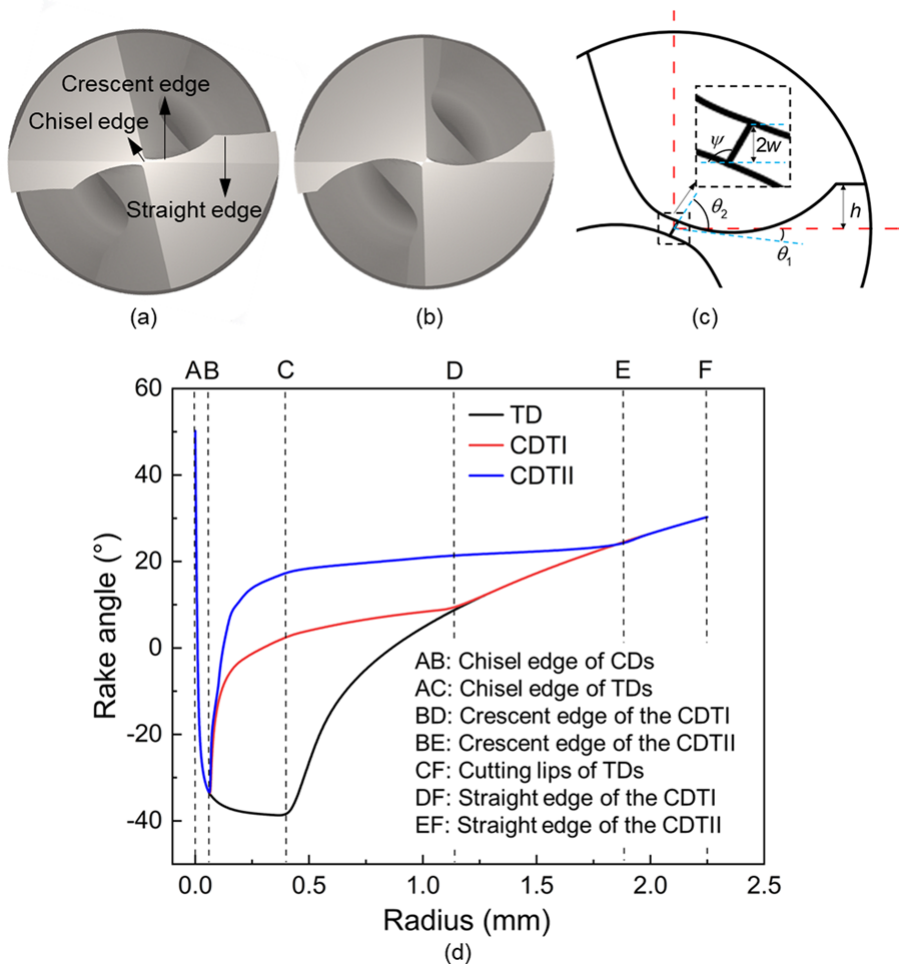
Design of CDs **a** Type I **b** Type II **c** schematic diagram of drill tip **d** Distribution of the rake angle of the crescent drill type I (CDTI), the crescent drill type II (CDTII) and the TD

Based on the results of the change in the chip area difference obtained, it is necessary to shorten the length of the drill’s chisel edge to reduce the difference in chip area, thereby suppressing the occurrence of skidding. The main purpose of this study was to verify the overall performance of the crescent drill, particularly its anti-skid properties, and to explore the influence of the drill bit structure on bone drilling. For this purpose, two types of crescent drills were designed with sharpened chisel edges, the crescent edges, and straight edges, as shown in Fig. 3. The crescent edge increases the rage angle, particularly converting a negative rake angle into a positive rake angle, as shown in Fig. 3d. The main differences between two crescent drills are the length and the polar angle $\theta$ of the crescent edge. The crescent drill type I (CDTI) has a large polar angle and a short length crescent edge, while the crescent drill type II (CDTII) has a small polar angle and a long length crescent edge.

### Experimental procedures

The workpiece material was collected from mid-diaphysis of bovine femurs in this study, as the distribution of its constituents is very similar to the human bone [20]. The bones were kept deep frozen to minimize changes in their mechanical properties. Before experiments, the bovine bone specimens were thawed, brought to room temperature, and fully immersed in saline solution until needed. And they were processed into 60(length) × 5(width) × 20(height) mm^3^ workpieces with smooth surfaces.

**Fig. 4:**
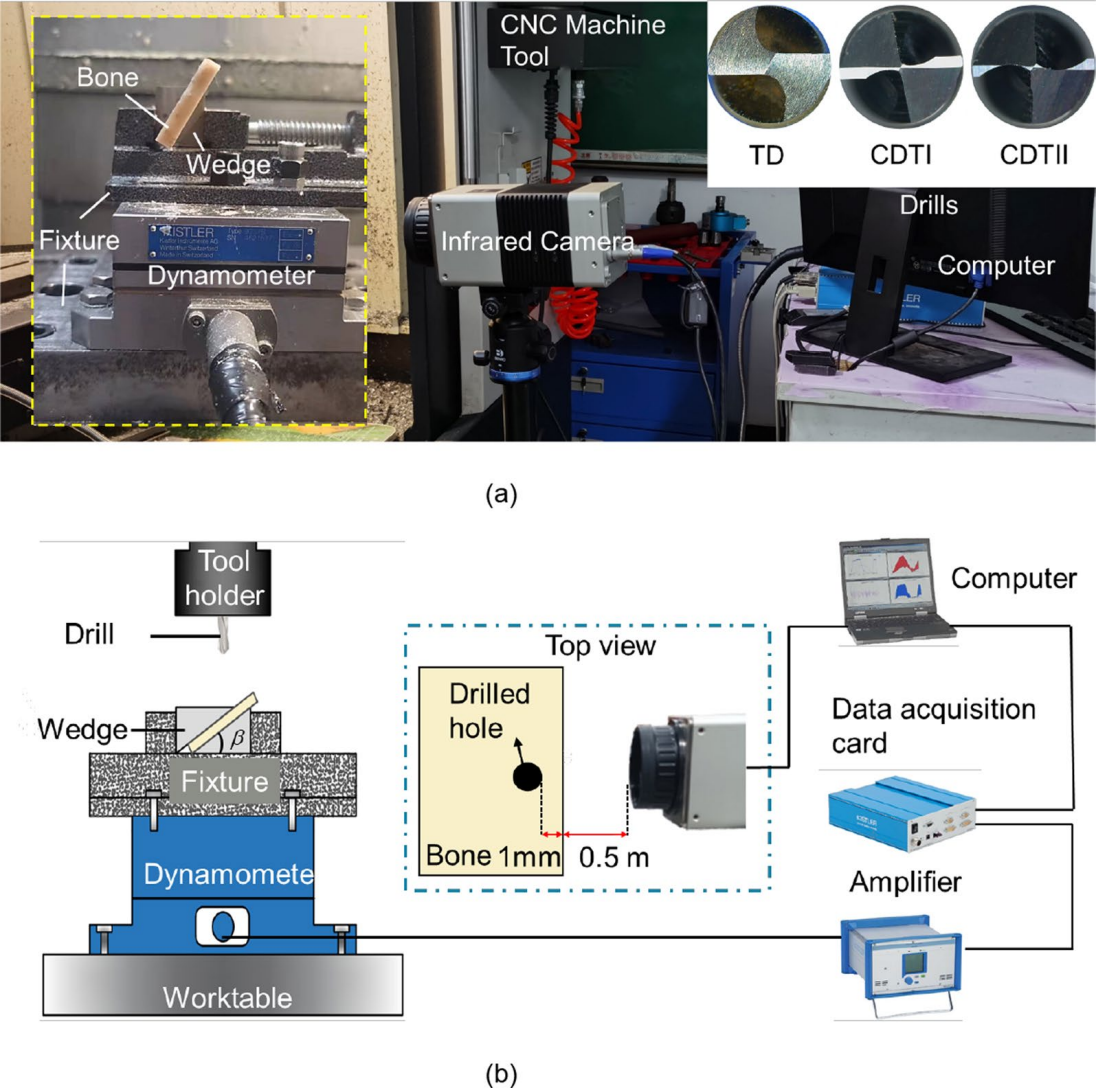
Inclined drilling operations of bones **a** experimental setup **b** schematic illustration of experimental platform

Figure 4a illustrates the experimental setup for the inclined drilling of bovine cortical bones. The drilling processes were conducted on a CNC milling machine (XK714D, Hanchuan CNC Machine Tool Co., Ltd., China). The bone workpiece was tilted and secured by wadges, and the workpiece and the horizontal plane set at angles of 0°, 15°, 30°, 45°, 60° ($\beta$). And the bone workpiece and dynamometer (9257A, Kistler Instrument Co., Ltd., Swiss) were fixed on the machine worktable with fixtures. The drilling processes were performed at a spindle speed *N* of 1200 rpm and feed rates *f* of 25 mm/min and 50 mm/min according to clinical statistics [21]. Throughout the drilling process, force data in three directions measured by the dynamometer was amplified by the charge amplifier (5070A, Kistler Instrument Co., Ltd., Swiss) and transmitted to the computer through the data acquisition card in real time. The force data were captured by the Dynoware software (Type 2825A-02, Kistler Instrument Co., Ltd., Swiss) with a sample rate of 10 kHz. The infrared camera (VarioCAM HD head 800, InfraTec GmbH, Germany) was performed to record the temperature at the hole wall. And the infrared camera is 0.5 m away from the workpiece. The emissivity of bone was set as 0.96 [22]. When observing the hole wall, it was situated 1 mm from the edge of the workpiece, as shown in Fig. 4b. To verify the effect of chisel edge length on skidding, we use drills with different chisel edge lengths (TDs, the CDTIs and the CDTIIs) for experimentation and comparative analysis of the skidding force and hole entrance morphology generated by them, thus correlating the area difference caused by changes in chisel edge length in Section “Analysis of drill skid” with experimental results. To further verify the effect of different inclination angles on skidding, this study conducts drilling experiments under different angles and compares and analyzes the skidding force and morphology generated by three types of drills at different angles. The drills used in this study is shown in Fig. 4a, including the TDs, the CDTIs and the CDTIIs. The drill specifications are listed in Table 1. The crescent drills used in this study can achieve “core thinned”. Compared with TD, the core thickness (2*w*) of the crescent drill bits is significantly reduced, while the web thickness (2*h*) remains the same. 3D Optical Profilometer (VR-6000, KEYENCE Co., Ltd., Japan) was used to observe the surface morphology of machined workpieces.

**Table 1:** Specification of drills used for the study

	CDTI	CDTII	TD
Diameter ($D$)	4.5 mm	4.5 mm	4.5 mm
Material	Cemented carbide	Cemented carbide	Cemented carbide
Point angle ($2\phi$)	118°	118°	118°
Helix angle ($\beta_{{\text{H}}}$)	30°	30°	30°
Chisel edge angle ($\psi$)	120°	120°	120°
Core thickness ($2w$)	0.11 mm	0.11 mm	0.76 mm
Web thickness ($2h$)	0.76 mm	0.76 mm	0.76 mm
Cutting edges	Straight edges, crescent edges, chisel edge	Straight edges, crescent edges, chisel edge	Cutting lips, chisel edge
Polar angle range of the crescent edge ($\theta_{1}$–$\theta_{2}$)	9°–60°	− 4°–60°	N/A

All experiments were conducted at room temperature (approximately 27 °C). To maintain the freshness of the bones, experiments were performed within an hour of preparation. Dry cutting conditions were employed throughout the experiment. At least three sets of experiments were carried out under each cutting condition, and the average values were considered for analysis. Sufficient time should be allowed between two drilling experiments to enable the workpiece and the drill to return to room temperature. And there is sufficient distance between the two holes. Replace the drill every hole to prevent the drill from wearing out.

## Results

### Skidding force and damage

**Fig. 5:**
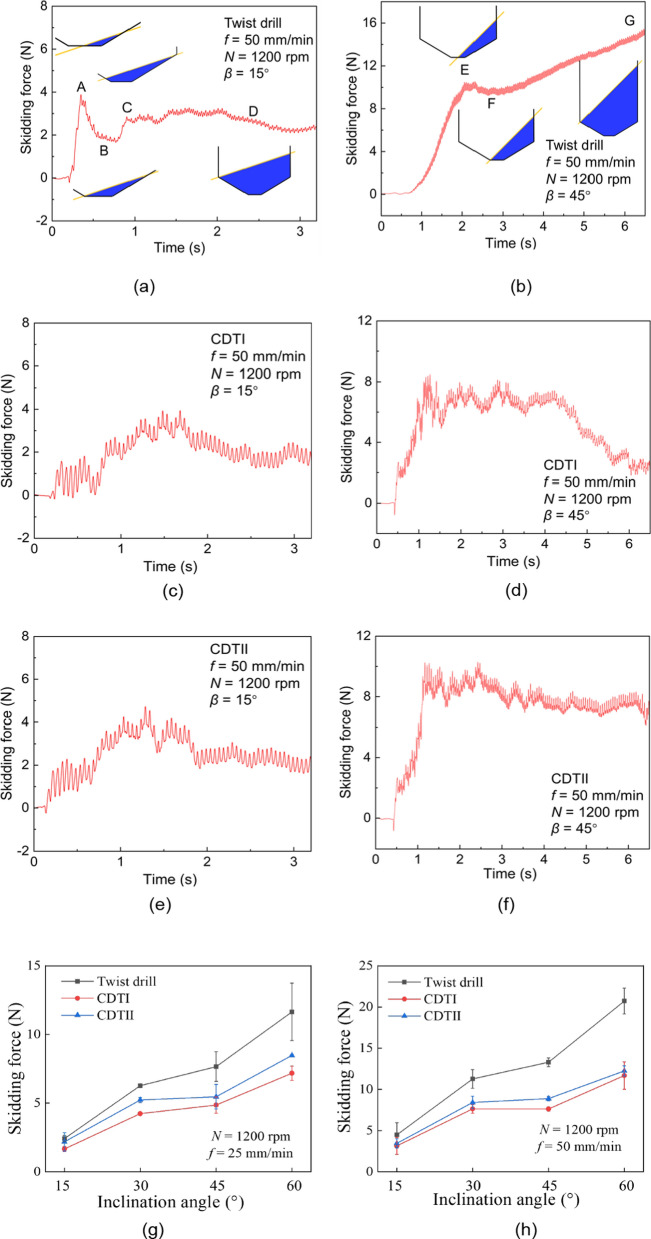
Curves of the skidding force $F_{X}$ at $\beta$ = 15° (**a**, **c**, **e**) and 45° (**b**, **d**, **f**) when the drill enters and skidding force under different drilling parameters **g** *f* = 25 mm/min (h) *f* = 50 mm/min

In this paper, the dynamometer monitors the drilling process of drill bits on inclined workpieces. The dynamometer can directly measure the horizontal component of the drilling force but cannot measure the component of the drilling forces parallel to the workpiece surface. Therefore, to maintain consistency between theoretical analysis and experimental research, the force measured on the X-axis $F_{X}$ by the dynamometer is defined as the skidding force. As a representation of small and large inclination angles respectively, Fig. 5 shows the $F_{X}$ when drills enter at *β* = 15° ($\beta < \frac{\pi }{2} - \phi$) and *β* = 45° ($\beta > \frac{\pi }{2} - \phi$). As can be seen in Fig. 5, the skidding force curves of the CDTI and the CDTII are smoother than that of the TD.

As shown in Fig. 5a, when $\beta < \frac{\pi }{2} - \phi$, according to the different drilling time, there are four key points in the TD entry process: (A) The midpoint of the chisel edge is engaged in cutting, and the other side of the drill is about to be engaged in cutting; (B) The chisel edge is fully engaged in cutting; (C) The lip on one side of the drill is fully engaged in cutting; (D) The drill tip enters the workpiece. As shown in Fig. 5b, when $\beta > \frac{\pi }{2} - \phi$, there are three key points in the drill entry process: (E) Similar to point A, the midpoint of the chisel edge is engaged in cutting and the other side of the drill is about to be engaged in cutting; (F) Similar to point B, the chisel edge is fully engaged in cutting; (G) Similar to point D, the drill tip enters the workpiece.

As shown in Fig. 5a, b, at points A and E, only one side of the drill is engaged in cutting, and the chip area difference reaches the maximum value when cutting on one side, so the skidding force is largest. As cutting continues, the chisel edge on the other side becomes engaged, and the radial force $F_{p}$ (see Fig. 1a) generated on both sides of the drill start to counterbalance each other. In addition, the chisel edge with negative rake angles generates a large $F_{p}$. At points B and F, where the $F_{p}$ generated by the chisel edge is all cancelled out, the skidding force reaches a valley value. And the skidding force then rises as the chip area difference reaches maximum at point C. At point D, the drill tip enters the workpiece completely, the chip area difference disappears, and the skidding force decreases slightly. In the FG section, the $F_{X}$ of the TD continues to rise.

The peak of the skidding force (in Fig. 5b, point E is regarded as the peak value) was selected for statistical analysis, as shown in Fig. 5g, h. It can be seen that at different inclination angles, the TD generates more skidding force than the CDTI and the CDTII. And the CDTI generates a minimal skidding force. Compared to CDTII, CDTI has a shorter length of the crescent edge, with a positive value of the polar angle *θ*_1_. This may result in a smaller component of the total drilling force being resolved in the horizontal direction, thereby leading to a minimal skidding force. At different feed rates, the skidding force increases as the inclination angle increases. This is the same as the conclusion in Fig. 2b. Obviously, the TD generates a significantly larger skidding force than the crescent drills at $\beta$ = 45° and 60°.

**Fig. 6:**
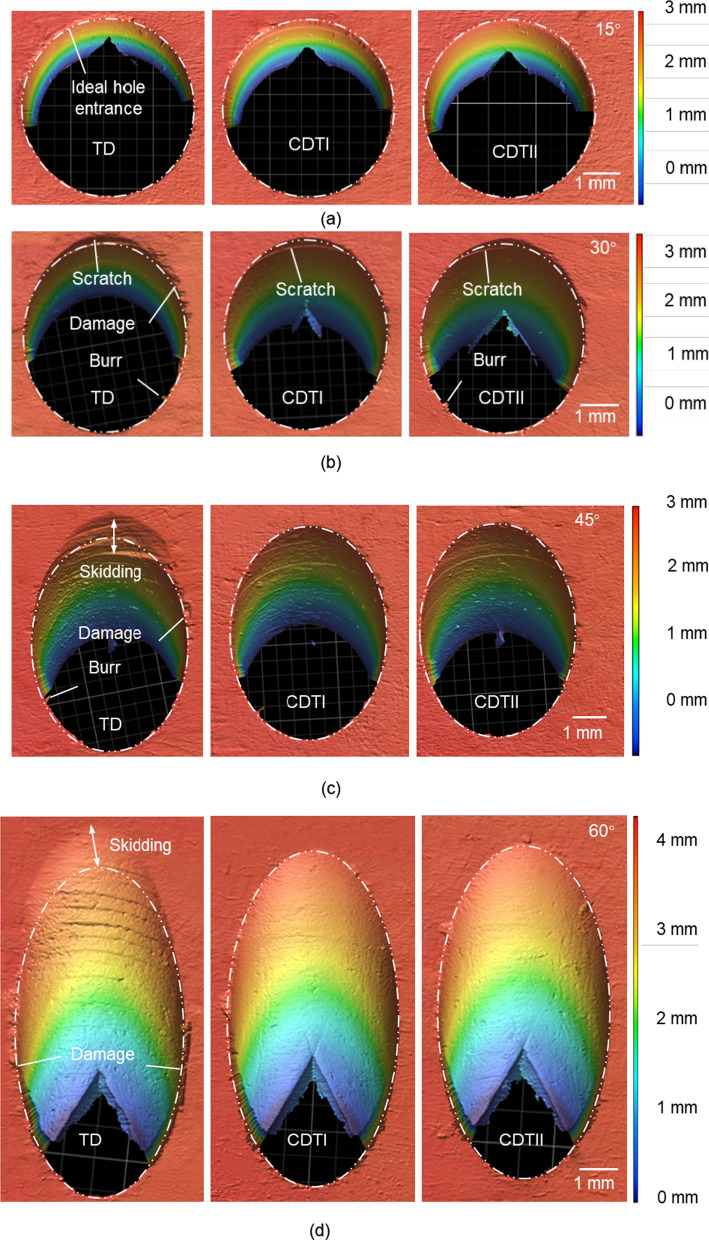
Images of the hole entrance and their ideal contours with inclination angles of 15°, 30°, 45° and 60°

The images of the hole entrance when *f* = 50 mm/min and *N* = 1200 rpm are shown in Fig. 6. When the inclination angle is equal to 15, the actual hole entrances are all consistent with the ideal hole entrances. No visible burrs or damage were observed. The processing quality of the hole entrance is good. When the inclination angle is equal to 30°, there are a few burrs, and the TD caused more serious damage to the tissue around the hole. In addition, a visible scratch can be observed in all three holes. When the inclination angle is equal to 45°, the TD generates holes with obvious scratches, poor quality walls, and severely damaged tissue around the hole. While only a few burrs and minor scratches and tissue damage are observed in the holes drilled by the CDTI and the CDTII. When the inclination angle is equal to 60°, the damage to the tissue around the hole is the most severe, and the TD leaves multiple clear scratches on the hole wall.

### Inclined drilling thrust force

**Fig. 7:**
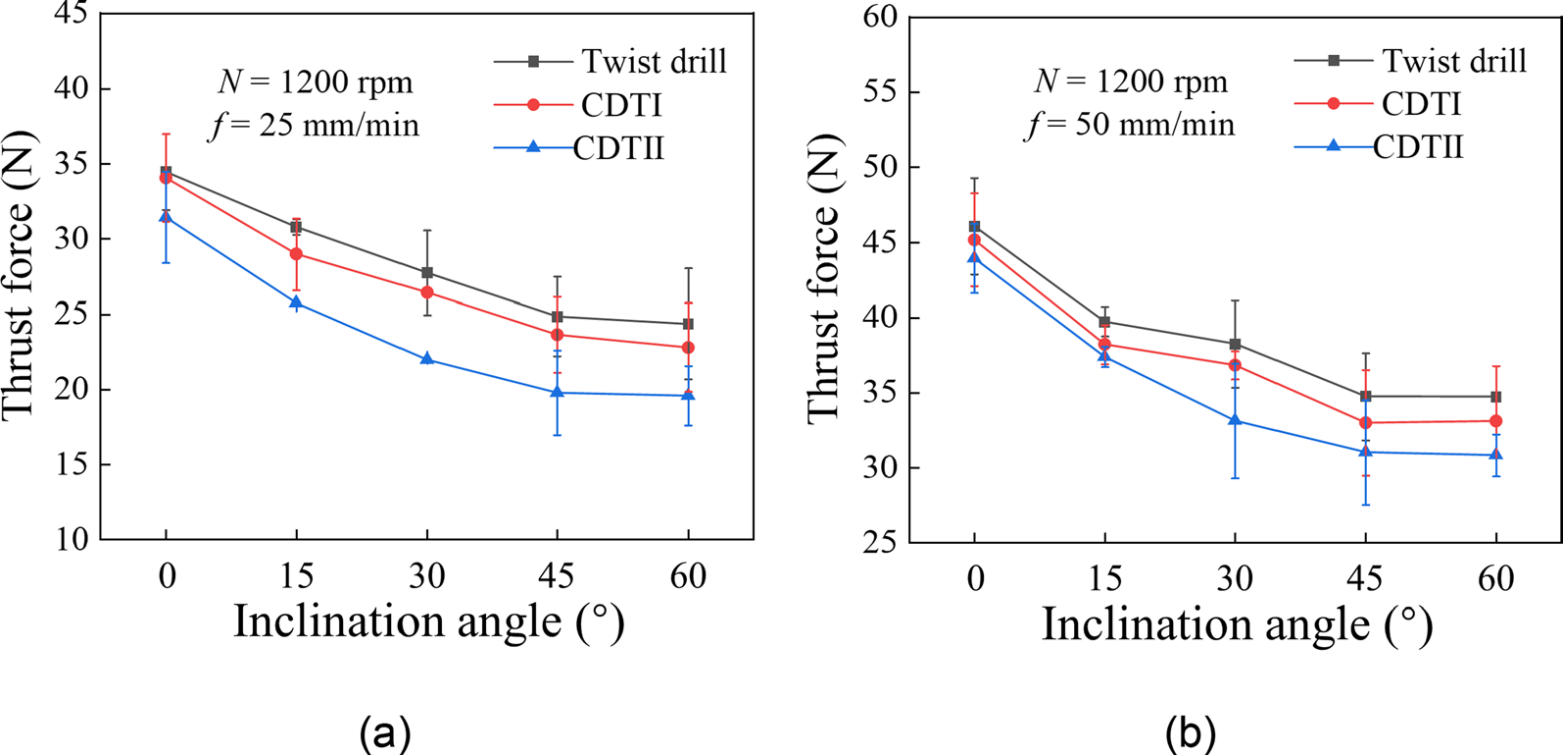
Thrust force under different drilling parameters **a** *f* = 25 mm/min **b** *f* = 50 mm/min

Figure 7 illustrates the thrust force at different inclination angles. The thrust force is taken from the stable drilling stage after the drill tip has fully entered the workpiece. It can be seen that the thrust force when drilling at an incline ($\beta \ne 0^\circ$) is less than the thrust force when drilling without an incline ($\beta = 0^\circ$). Both crescent drills generate a smaller thrust force than the TD, and the CDTII generates the minimum thrust force at all inclination angles. In addition, due to the inherent structural characteristics of bone materials, local hardness variations may exist in bone specimens. When the drill bit encounters regions that are harder or tougher at different inclination angles, it may require additional thrust force for drilling. Consequently, an increase in thrust force was observed at individual inclination angles. However, the overall trend of this study still indicates that thrust force decreases with an increase in inclination angle.

### Drilling temperature

**Fig. 8:**
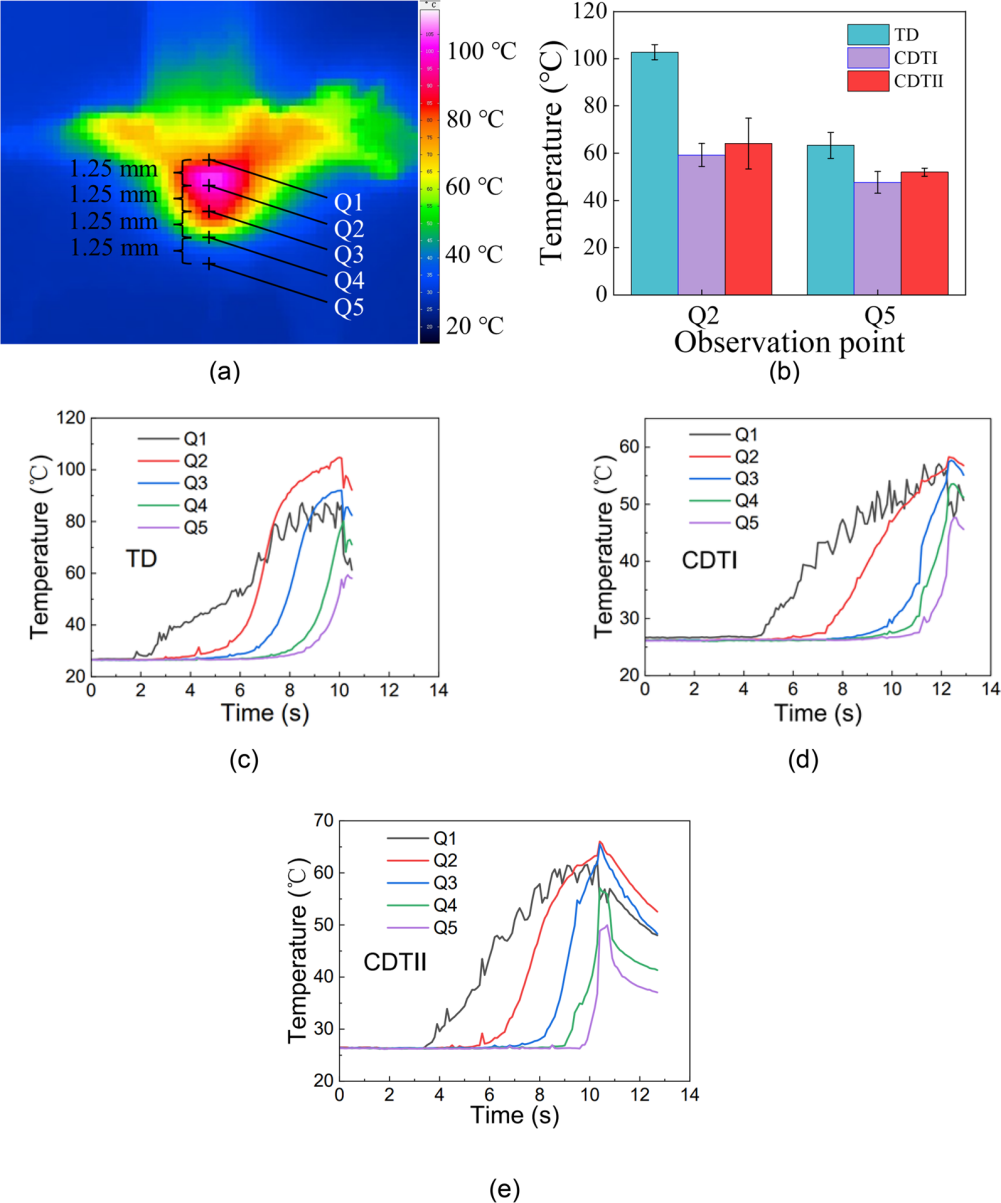
Analysis of drilling temperature **a** Thermal imaging and data acquisition of the bone drilling **b** Maximum temperatures of the hole wall at Q2 and Q5 **c** Temperature generated by the TD **d** Temperature generated by the CDTI **e** Temperature generated by the CDTII

Five observation points Q1–Q5 on the hole wall were set up to observe the temperature variation at different depths during drilling when *f* = 50 mm/min and *N* = 1200 rpm. The distance between the two points is 1.25 mm, as shown in Fig. 8a. Where Q1 is the upper surface of the workpiece and Q5 is the lower surface of the workpiece. Figure 8b shows the maximum temperature of the hole wall at Q2 (close to the entrance) and Q5 (hole exit). Figure 8c, d, e illustrates the temperature variations of the hole wall at different depths.

As shown in Fig. 8b, at different observation points, the TD generated the highest temperature, the CDTII the second highest and the CDTI the lowest. In the process of inclined drilling, the skidding force is one of the main factors affecting drilling temperature. Compared to CDTII, CDTI has lower skidding force. A decrease in skidding force implies reduced friction between the drill bit and the workpiece surface, resulting in lower heat generation and consequently reduced drilling temperature. Therefore, the temperature of CDTI is lower than that of CDTII. In addition, the highest temperature position in the process of the bone drilling is not the hole exit. Figure 8c, d, e also shows the same result. During drilling, the maximum temperature generated by the TD is 79.82% higher than that of the CDTI and 58.69% higher than that of the CDTII. It can be found from Fig. 8 that the temperature is the highest in the middle of the hole wall (Q2, Q3), followed by the hole entrance, and the hole exit temperature is the lowest.

## Discussion

In this study, a chip area difference model for $\beta < \frac{\pi }{2} - \phi$ and $\beta > \frac{\pi }{2} - \phi$ was first proposed. Two types of crescent drills were developed to reduce thrust force and drilling temperature and prevent drill skidding in orthopedic surgery. Inclined bone drilling experiments were conducted to evaluate the performance of the crescent bone drills. Temperature variations at the hole wall drilled with different drills were observed using an infrared camera.

Drill skidding can be inferred by the $F_{X}$ and the drilling damage. When $\beta < \frac{\pi }{2} - \phi$, the increase of inclination angle leads to the increase of the chip area difference, which leads to the increase of the $F_{X}$. When $\beta > \frac{\pi }{2} - \phi$, skid and drill bending significantly affect the $F_{X}$. When $\beta$ = 15°, none of the three drills skidded. When $\beta$ = 30°, all three drills skidded slightly. This may be because the inclination angle is approximately equal to $\frac{\pi }{2} - \phi$ (31°), and the whole main cutting-edge contacts the workpiece almost at the same time, which reduces the anti-skid ability of the drill. This phenomenon indicates that the inclination angle should avoid $\frac{\pi }{2} - \phi$. When $\beta$ = 45° or 60°, severe skid occurs in the TD, while no skid is observed in the CDs, which illustrates the good performance of the crescent edge in anti-skid. In the FG section of Fig. 5b, the drill skidded and bent severely, with bending dominating the skidding force. The relationship between the $F_{X}$ and the resulting deflection $w_{\theta }$ can be expressed by $w_{\theta } = F_{X} l^{3} /3EI$, where *EI* is the flexural rigidity and *l* is the length of the drill cantilever. In the FG section, as the drill feeds, the bending becomes more severe, the deflection of the drill tip $w_{\theta }$ increases, and the corresponding skidding force $F_{X}$ increases.

In general, bone drilling produces little burr while causing damage to the surrounding tissue. Increasing the inclination angle increases the skidding of the drill, resulting in an increase in the skidding distance and more scratches. This makes the contour of the hole entrance more different from the ideal contour and results in a poorer quality finish on the hole wall. Furthermore, increasing the inclination angle increases the damage to the tissue around the hole. The drilling quality of the two crescent drills is better than that of the TD, and crescent drills generate less damage. There is no obvious difference in the drilling quality of the two crescent drills. This is because both crescent drills have excellent anti-skid capabilities and are significantly better than TDs.

Most academics currently use thermocouples to capture drilling temperature or thermal imaging cameras to capture hole exit temperature, but little attention has been paid to the location where the highest temperatures are generated [18, 19, 23]. First, the temperature at the hole entrance is not the highest. Although the wall-drill friction time experienced at the hole entrance is the longest, the heat dissipation conditions are good. And the airflow generated by the rotation of the drill at the hole entrance takes away heat, causing fluctuations in the temperature curve at Q1. The position close to the hole entrance (Q2) experiences a long friction time. Due to the low thermal conductivity of cortical bone and poor heat dissipation conditions, heat accumulates at Q2, resulting in the highest temperature readings. The heat dissipation conditions at the hole exit are also good, and the friction time experienced here is the shortest. Therefore, the temperature at the hole exit is the lowest. Therefore, the temperature information obtained by monitoring the hole wall of the bone drilling is more accurate.

In comparison, the CDs outperform the TD in all aspects of bone drilling. The CDTI performs better in reducing the skidding force and temperature, while the CDTII performs better in reducing the thrust force. As shown in Fig. 3d, the small and negative polar angle of the CDTII gives its crescent edges a larger rake angle, which leads to a reduction in thrust forces. Combined with Fig. 5, the CDTII generates a larger $F_{X}$, so the small and negative polar angle may allow the total drilling force to be decomposed more into the horizontal direction. However, no optimal solution has yet been derived for the length and polar angle of the crescent edge. Our future work will determine the optimal tip geometry of the crescent drill and explore new bone drill structures.

## Conclusions

In this study, the effects of drill geometry parameters on bone drilling thrust forces, chip deformation, temperature and skidding were analyzed and a chip area difference model for $\beta < \frac{\pi }{2} - \phi$ and $\beta > \frac{\pi }{2} - \phi$ was first proposed. Two types of crescent drills were designed to reduce the thrust force and drilling temperature and prevent drill skidding in orthopedic surgery. Orthogonal bone cutting experiments and inclined bone drilling experiments were conducted to evaluate the crescent bone drills. Some conclusions of this study are as follows:

1. Two types of crescent drills were designed with the sharpened chisel edge, the crescent edge, and the straight edge. The crescent edge enhances the rage angle, notably converting a negative rake angle into a positive rake angle. The crescent drill type I (CDTI) has a large polar angle and a short length, while the crescent drill type II (CDTII) has a small polar angle and a long length.
2. The results of the orthogonal bone cutting experiments showed that increasing the rake angle, especially by converting a negative rake angle to a positive rake angle, can effectively reduce the cutting force and thrust force. When compared to a cutting tool with a − 30° rake angle, a cutting tool with 20° rake angle generated more than 49.64% less cutting force and more than 59.88% less thrust force.
3. Lengthening the chisel edge length and increasing the inclination angle of the workpiece will amplify the difference in chip area and result in more pronounced skidding. Inclined bone drilling experiments were carried out. The results showed that the TD had the worst drilling quality and the TD skidded and bent at large inclination angles (45° and 60°). The crescent drills did not skid and caused little drilling damage. In comparison, the CDTI performs better in reducing skidding force, whereas CDTII excelled in reducing thrust force.
4. Temperature variations at the hole wall drilled with different drills were observed with an infrared camera. The highest temperature was observed in the middle of the hole wall because of the prolonged workpiece-drill friction and poor heat dissipation conditions. The TD generates the highest temperature, followed by the CDTII and the CDTI generates the lowest temperature.
5. According to experimental results, a longer crescent edge with a small and negative polar angle can increase the rake angle of the tool, thus reducing the thrust force. And this also increases the skidding force and temperature.

## Data Availability

No datasets were generated or analysed during the current study.
